# Anticoagulantes Orais Diretos Ininterruptos em Ablação por Cateter de Fibrilação Atrial: Pronto para a Prática Clínica

**DOI:** 10.36660/abc.20200110

**Published:** 2020-04-06

**Authors:** Rhanderson Cardoso, André D’Avila

**Affiliations:** 1 Divisão de CardiologiaJohns Hopkins HospitalBaltimoreMarylandEUADivisão de Cardiologia - Johns Hopkins Hospital, Baltimore, Maryland - EUA; 2 Serviço de Arritmia CardíacaHospital SOS CardioFlorianópolisSCBrasilServiço de Arritmia Cardíaca - Hospital SOS Cardio, Florianópolis, SC - Brasil

**Keywords:** Fibrilação Atrial, Anticoagulantes, Ablação por Cateter, Rivaroxabana/uso terapêutico

A ablação por cateter é uma estratégia eficaz, segura e bem estabelecida para alcançar o controle do ritmo em pacientes com fibrilação atrial (FA) sintomática que são ou intolerantes ou refratários ao controle do ritmo farmacológico ou que desejam evitar o uso prolongado de drogas antiarrítmicas. Historicamente, quando os antagonistas de vitamina K (AVKs) eram a única opção para a anticoagulação oral, realizava-se a ablação por cateter após a interrupção do AVK durante vários dias e uma transição (ponte) para anticoagulação subcutânea ou parenteral, tipicamente com heparina de baixo peso molecular. Tal estratégia, porém, era inconveniente e cheia de complicações hemorrágicas. Além disso, o ensaio randomizado COMPARE e estudos observacionais demonstraram que o risco tromboembólico era de 10 a 15 vezes mais alto com AVK e ponte de heparina em comparação com AVK ininterrupto.^1^ Após esses resultados, o AVK ininterrupto com razão normalizada internacional (RNI) terapêutica se tornou padrão para anticoagulação periprocedural, e pacientes rotineiramente eram submetidos à ablação por cateter com RNI entre 2 e 3,5.

Essa opção, no entanto, também possui duas desvantagens importantes. A primeira é que a ablação se torna contingente ao RNI terapêutico no dia do procedimento. Um RNI supra-terapêutico pode implicar a decisão de adiar o procedimento ou administrar produtos sanguíneos, enquanto um RNI sub-terapêutico tipicamente implicaria adiar a ablação para outro dia ou requerer heparina IV até alcançar o RNI ideal. A segunda, o uso ininterrupto de AVKs entra em choque com o crescente uso de anticoagulantes orais diretos (AODs). Os eletrofisiologistas que programam ablação por cateter para pacientes sob uso de AODs devem enfrentar a seguinte decisão: (1) fazer transição para AVKs, para anticoagulação periprocedural ininterrupta ou (2) continuar AOD periprocedural.

Esta questão importante foi abordada por Silva et al.,^2^nesta edição de Arquivos Brasileiros de Cardiologia. Eles compararam 130 pacientes consecutivos com FA submetidos à ablação por cateter em um único centro sob uso ininterrupto de rivaroxabana com 110 pacientes de um grupo de controle histórico que havia previamente sido submetido à ablação por cateter sob uso ininterrupto de AVK com RNI pré-procedimento entre 2 e 3,5. Sangramento maior ocorreu em 1 (0.7%) e 2 (1.8%) pacientes nos grupos rivaroxabana e AVK, respectivamente. O evento no grupo rivaroxabana foi um hematoma retroperitoneal requerendo drenagem cirúrgica, e no grupo AVK houve um caso de hematoma femoral que foi tratado de modo conservador e um de efusão pericárdica requerendo pericardiocentese. Um paciente teve um acidente vascular cerebral isquêmico no grupo rivaroxabana (0.7%), enquanto não houve eventos tromboembólicos com AVKs.

Outros estudos, incluindo ensaios randomizados com todos os quatro AODs (rivaroxabana, apixabana, edoxabana e dabigatrana), têm chegado a conclusões semelhantes. Em uma meta-análise incluindo 12 estudos e quase 5.000 pacientes tratados com AVKs ou AODs ininterruptos, a incidência de acidente vascular cerebral acidente vascular e ataque isquêmico transitório periprocedurais foi baixa, e não foi significativamente diferente entre os dois grupos (AOD 0,08%, AVK 0,16%).^3^ Em um sub-coorte de pacientes submetidos a imagem cerebral pós-procedimento de rotina, a incidência de eventos embólicos clinicamente silenciosos também não foi significativamente diferente entre os dois grupos (AOD 8%; AVK 9,6%; RC 0,86; IC 95% 0,42 – 1,76). Houve uma incidência menor de sangramento maior nos pacientes que receberam AODs (0,9%) do que nos anticoagulados com AVKs (2%) (RC 0,50; IC 95% 0,30 – 0,84; p < 0,01). Não houve diferença entre os grupos quanto à ocorrência de tamponamento pericárdico (0,7% vs. 0,8% com AODs e AVKs, respectivamente).^3^

Ao todo, temos aprendido várias lições do estudo de Silva et al.,^2^ e de estudos semelhantes na literatura. Primeiramente, a incidência de acidente vascular cerebral periprocedural com uso ininterrupto de AOD é extremamente baixa, bem inferior a 1%, e semelhante à incidência com AVKs ininterruptos. Isso representa uma grande melhoria em comparação à estratégia histórica de interromper a anticoagulação oral com uma ponte de heparina, onde a incidência de eventos tromboembólicos variava de 1% a 5%.^1^ Não se pode exagerar a importância desse achado. Uma baixa incidência de eventos tromboembólicos é fundamental para o tratamento da FA por ablação por cateter, que é um procedimento indicado quase exclusivamente para controlar sintomas e não com o fim de salvar vidas. Vale ressaltar que o significado clínico da embolia cerebral assintomática em pacientes submetidos à ablação por cateter ainda não está esclarecido neste momento. Estudos futuros deverão examinar os desfechos clínicos e a função cognitiva a longo prazo nos pacientes que apresentam eventos embólicos cerebrais clinicamente silenciosos.

Em segundo lugar, a incidência de complicações hemorrágicas graves com AODs ininterruptos também é baixa e comparável, se não melhor, à incidência com AVKs ininterruptos. No presente estudo, um cálculo de potência, com alfa bilateral de 0,05 e taxa de eventos de 2,5% no grupo controle, produziria uma potência estimada de apenas 3% para detectar uma diferença de 1% nos eventos de sangramento maior entre os grupos com o tamanho da amostra de 240 pacientes. Isso, no entanto, não deve ser visto como uma limitação do estudo, mas como uma demonstração da segurança do procedimento tanto com AVKs quanto com AODs. Da mesma forma, dois grandes ensaios randomizados, o VENTURE-AF (rivaroxabana) e o RE-CIRCUIT (dabigatrana), incluindo 248 e 704 pacientes, respectivamente, reconheceram estar com potência inadequada para o desfecho primário de sangramento maior.^4,5^

Previamente, a apreensão quanto à falta de reversibilidade dos AODs limitava a ampla aceitação dessa estratégia. Essa preocupação tem diminuído em grande parte devido ao desenvolvimento da idarucizumab e andexanet alfa, agentes de reversão para dabigatrana e inibidores de fator Xa, respectivamente. Mais importante, talvez, é o fato que a estratégia geral de AODs ininterruptos provou ser muito segura, com uma baixa incidência de eventos de sangramento maior. No RE-CIRCUIT, embora a idarucizumab estivesse disponível, não foi necessária em nenhum dos 317 pacientes submetidos à ablação por cateter sob uso de dabigatrana ininterrupta, incluindo uma dose administrada na manhã da ablação.^5^ Em uma análise conjunta de 14 pacientes com tamponamento cardíaco de 3 estudos randomizados de AODs vs. AVKs ininterruptos, todos foram submetidos à pericardiocentese; 12 receberam protamina, e 2 (no grupo AVK) receberam concentrado de complexo de protrombina. Nenhum paciente recebeu um agente de reversão direta de AOD.^6^

Eventos hemorrágicos também podem ser evitados com atenção meticulosa à hemostasia. O uso de uma sutura em oito para o fechamento venoso em pacientes totalmente anticoagulados ao final do procedimento também tem o potencial de diminuir a formação de hematoma e diminuir a duração do repouso após a ablação por cateter.^7^ Essa sutura hemostática pode evitar a necessidade de reversão da protamina, estendendo a anticoagulação terapêutica durante as horas após o procedimento. Merece investigação futura se essa técnica reduz o risco tromboembólico (já baixo), com uma incidência aceitável de eventos hemorrágicos.

Finalmente, é importante enfatizar a distinção entre uma estratégia verdadeiramente ininterrupta, na qual o AOD é administrado durante o pré-procedimento no horário e na dose usuais, e uma estratégia minimamente interrupta alternativa, na qual o AOD é interrompido por 1 ou 2 doses antes da ablação por cateter. Em ambas estratégias, o AOD é tipicamente reiniciado no mínimo 4 horas após retirar a bainha venosa femoral. Esse é um dilema particular com agentes tomados duas vezes por dia, em que é necessário tomar uma decisão sobre a dose matinal do AOD no dia da ablação; é menos preocupante com agentes de uma dose diária, como rivaroxabana, onde a droga pode ser administrada ininterruptamente à noite antes da ablação por cateter, sem requerer uma dose matinal. No ensaio ABRIDGE-J, 504 pacientes encaminhados para ablação por cateter de FA foram randomizados para dabigatrana minimamente interrupta (evitando-se 1 ou 2 doses pré-procedimento) ou AVKs ininterruptos. Não houve eventos tromboembólicos nos 220 pacientes submetidos à ablação no grupo dabigatrana. A dabigatrana minimamente interrupta foi associada a uma menor incidência de sangramento maior (1,4%) em comparação com AVKs ininterruptos (5%).^8^ Deve-se enfatizar, no entanto, que, embora haja dados robustos e consistentes apoiando uma estratégia de AODs ininterruptos para anticoagulação em pacientes submetidos à ablação por cateter de FA, o uso de uma estratégia minimamente interrupta não foi extensivamente estudado nem diretamente comparado ao uso ininterrupto de AODs em grandes estudos randomizados.

Em conclusão, estudos têm demonstrado que a anticoagulação ininterrupta com AOD para pacientes submetidos à ablação por cateter de FA é eficaz na prevenção de eventos tromboembólicos periprocedurais (< 1%). Essa estratégia também tem um baixo risco de eventos de sangramento maior, comparável ou inferior aos eventos hemorrágicos com AVKs ininterruptos. Espera-se que estudos prospectivos na área investiguem abordagens mecânicas para minimizar eventos hemorrágicos e avaliem a eficácia e a segurança de uma estratégia minimamente interrupta de AODs. Até então, o uso ininterrupto de AODs deve prevalecer como a opção de anticoagulação preferencial para pacientes submetidos à ablação por cateter. Os autores devem ser parabenizados por sua iniciativa e pelo estudo bem conduzido.
