# Supplementary material for: Anticoagulantes Orais Diretos Ininterruptos em Ablação por Cateter de Fibrilação Atrial: Pronto para a Prática Clínica
Source: Arq Bras Cardiol. 2020 Apr 6;114(3):443–5. [Article in Portuguese] doi: 10.36660/abc.20200110 (PMC7792720; doi:10.36660/abc.20200110)
Supplement: Translation

# Translation: Uninterrupted Direct Oral Anticoagulants in Atrial Fibrillation Catheter Ablation: Ready for Prime Time

**Authors:** Rhanderson Cardoso, André D’Avila

**Affiliations:** 1 USADivisão de Cardiologia - Johns Hopkins Hospital, Baltimore, Maryland - USA; 2 BrazilServiço de Arritmia Cardíaca - Hospital SOS Cardio, Florianópolis, SC - Brazil

**Keywords:** Atrial Fibrillation, Anticoagulants, Catheter Ablation, Rivaroxaban/therapeutic use

Catheter ablation is a well-established, safe, and effective strategy to achieve rhythm control in patients with symptomatic atrial fibrillation (AF) who are either intolerant or refractory to pharmacologic rhythm control or who wish to avoid long-term use of anti-arrhythmic drugs. Historically, when vitamin-K antagonists (VKAs) were the only option for oral anticoagulation, catheter ablation was performed after interruption of the VKA for several days and a transition (bridge) to subcutaneous or parenteral anticoagulation, typically with low-molecular-weight heparin. This strategy, however, was cumbersome and fraught with bleeding complications. Furthermore, the COMPARE randomized trial and observational studies showed that the thromboembolic risk was 10 to 15-fold higher with VKAs and heparin bridging as compared to uninterrupted VKAs.^1^ After these results, uninterrupted VKAs with a therapeutic international normalized ratio (INR) became the standard of care for periprocedural anticoagulation, and patients would routinely undergo catheter ablation with INR ranging between 2 and 3.5.

This option, however, also has two important setbacks. First, ablation becomes contingent on a therapeutic INR on the day of the procedure. A supra-therapeutic INR may entail a decision to postpone the procedure or administer blood products for correction, whereas a sub-therapeutic INR would typically imply deferring ablation to another day or require IV heparin until an ideal INR is reached. Second, the use of uninterrupted VKAs conflicts with the ever growing use of direct oral anticoagulants (DOACs). Electrophysiologists planning catheter ablation for patients on DOACs are faced with the following decision: (1) transition to VKAs for uninterrupted periprocedural anticoagulation or (2) continue periprocedural DOAC.

This important question was addressed by Silva et al.^2^ in this issue of the Brazilian Archives of Cardiology. They compared 130 consecutive patients with AF who underwent catheter ablation in a single center while receiving uninterrupted rivaroxaban to 110 patients in a historic control group who had previously undergone catheter ablation on uninterrupted VKA with a pre-procedure INR between 2 and 3.5. Major bleeding occurred in 1 (0.7%) and 2 (1.8%) individuals in the rivaroxaban and VKA groups, respectively. The event in the rivaroxaban group was a retroperitoneal hematoma requiring surgical drainage. In the VKA group, there was a femoral hematoma treated conservatively and a pericardial effusion requiring pericardiocentesis. One patient had an ischemic stroke in the rivaroxaban group (0.7%), while there were no thromboembolic events with VKAs.

Other studies, including randomized trials with all four DOACs (rivaroxaban, apixaban, edoxaban, and dabigatran), have reached similar conclusions. In a meta-analysis including 12 studies and nearly 5,000 patients treated with uninterrupted VKAs or DOACs, the incidence of periprocedural stroke or transient ischemic attack was low, and it was not significantly different between the two groups (DOAC 0.08%, VKA 0.16%).^3^ In a sub-cohort of patients who underwent routine post-procedure brain imaging, the incidence of clinically silent embolic events was also not significantly different between both groups (DOAC 8%; VKA 9.6%; OR 0.86; 95% CI 0.42 – 1.76). There was a lower incidence of major bleeding in those who received DOACs (0.9%) than in patients anticoagulated with VKAs (2%) (OR 0.50; 95% CI 0.30 – 0.84; p < 0.01). There was no difference between groups in the occurrence of pericardial tamponade (0.7% vs. 0.8% with DOACs and VKAs, respectively).^3^

Altogether, we have learned several lessons from the study by Silva et al.^2^ and similar studies in the literature. First, the incidence of periprocedural stroke with uninterrupted DOAC use is exceedingly low, well under 1%, and similar to that of uninterrupted VKAs. This represents a major improvement compared to the historic strategy of interrupting oral anticoagulation with a heparin bridge, where the incidence of thromboembolic events ranged from 1% to 5%.^1^ The importance of this finding cannot be overstated. A low incidence of thromboembolic events is paramount when treating AF by catheter ablation, a procedure that is indicated almost exclusively for symptom control and not for life-saving purposes. It is noteworthy that the clinical significance of asymptomatic cerebral embolism in patients who undergo catheter ablation is unclear at this point. Further studies should examine long-term clinical outcomes and cognitive function in those who have clinically silent cerebral embolic events.

Second, the incidence of major hemorrhagic complications with uninterrupted DOACs is also low, and it is comparable to, if not better than, that of uninterrupted VKAs. In the present study, a power calculation, with two-sided alpha of 0.05 and a 2.5% event rate in control group, would yield an estimated power of only 3% to detect a 1% difference in major bleeding events between groups with the sample size of 240 patients. This, however, should not be viewed as a limitation to the study, but rather as a testament to the safety of the procedure with both VKAs and DOACs. Similarly, two large randomized trials, VENTURE-AF (rivaroxaban) and RE-CIRCUIT (dabigatran), including 248 and 704 patients, respectively, acknowledged being underpowered for their primary endpoint of major bleeding.^4,5^

Previously, apprehension regarding the lack of reversibility of DOACs limited widespread acceptance of this strategy. This concern has largely abated with the development of idarucizumab and andexanet alpha, reversal agents for dabigatran and factor Xa inhibitors, respectively. More importantly, perhaps, is that the overall strategy of uninterrupted DOACs has proven to be very safe with a low incidence of major bleeding events. In RE-CIRCUIT idarucizumab, although it was available, was not required in any of the 317 patients who underwent catheter ablation while on uninterrupted dabigatran, which included a dose administered on the morning of ablation.^5^ In a pooled analysis of 14 patients with cardiac tamponade from 3 randomized trials of uninterrupted DOACs vs. VKAs, all underwent pericardiocentesis; 12 received protamine; and 2 (in the VKA group) received prothrombin complex concentrate. None received a direct DOAC reversal agent.^6^

Bleeding events can also be prevented by meticulous attention to hemostasis. The use of a figure-of-eight suture for venous closure in patients who are fully anticoagulated at the end of the procedure also has the potential to decrease hematoma formation and shorten bedrest duration after catheter ablation.^7^ This hemostatic suture may obviate the need for protamine reversal, extending therapeutic anticoagulation during the hours following the procedure. Whether this technique further reduces the (already low) thromboembolic risk with an acceptable incidence of bleeding events warrants further investigation.

Finally, it is important to highlight the distinction between a truly uninterrupted strategy, where the DOAC is given pre-procedurally at the usual time and dose and an alternative minimally interrupted strategy, where 1 or 2 doses of the DOAC are held prior to catheter ablation. In both strategies, the DOAC is typically resumed at a minimum of 4 hours after femoral venous sheath removal. This is a particular dilemma with twice-daily agents, where a decision has to be made about the morning DOAC dose on the day of ablation; it is less of a concern with once-daily options, such as rivaroxaban, where the drug can be administered uninterruptedly in the evening prior to catheter ablation, without requiring a morning dose. In the ABRIDGE-J trial, 504 patients scheduled for AF catheter ablation were randomized to minimally interrupted dabigatran (holding 1 to 2 pre-procedure doses) or uninterrupted VKAs. There were no thromboembolic events in the 220 patients who underwent ablation in the dabigatran group. Minimally interrupted dabigatran was associated with a lower incidence of major bleeding (1.4%) as compared to uninterrupted VKAs (5%).^8^ It should be emphasized, however, that while there is robust and consistent data supporting a strategy of uninterrupted DOACs for anticoagulation in patients undergoing AF catheter ablation, the use of a minimally interrupted strategy has neither been extensively studied nor directly compared to uninterrupted DOAC use in large randomized studies.

In conclusion, studies have demonstrated that uninterrupted anticoagulation with DOACs for patients undergoing AF catheter ablation is effective in the prevention of periprocedural thromboembolic events (< 1%). This strategy also has a low risk of major bleeding events, comparable to or lower than bleeding events with uninterrupted VKAs. Prospective studies in the field will hopefully investigate mechanical approaches to minimize bleeding events and evaluate the efficacy and safety of a minimally interrupted DOAC strategy. Until then, the use of uninterrupted DOACs should be strongly favored as the preferred anticoagulation option for patients undergoing AF catheter ablation. The authors should be congratulated for their initiative and well-conducted study.
